# Metabolic and genotypic characterization of meropenem-susceptible and meropenem-resistant *Serratia marcescens* isolates

**DOI:** 10.55730/1300-0144.6055

**Published:** 2025-06-29

**Authors:** Şeyma NİGİZ, Gülşen HAZIROLAN, Gülşen ALTINKANAT GELMEZ, Ceren ÖZKUL, Engin KOÇAK, Sevilay ERDOĞAN KABLAN, Emirhan NEMUTLU, Aycan GÜNDOĞDU, Fatma BAYRAKDAR, Ufuk HASDEMİR, Deniz GÜR

**Affiliations:** 1 Department of Pharmaceutical Microbiology, Faculty of Pharmacy, Hacettepe University, Ankara, Turkiye; 2 Department of Medical Microbiology, Faculty of Medicine, Hacettepe University, Ankara, Turkiye; 3 Department of Medical Microbiology, Faculty of Medicine, Marmara University, İstanbul, Turkiye; 4 Department of Analytical Chemistry, Gulhane Faculty of Pharmacy, University of Health Sciences, Ankara, Turkiye; 5 Department of Analytical Chemistry, Faculty of Pharmacy, Hacettepe University, Ankara, Turkiye; 6 Department of Medical Microbiology, Faculty of Medicine, Erciyes University, Kayseri, Turkiye; 7 Department of VetMEd-Infectious Diseases, College of Veterinary Medicine, University of Georgia, GA, USA

**Keywords:** *Serratia marcescens*, carbapenemases, microdilution, efflux pumps, pulsed-field gel electrophoresis, metabolomics

## Abstract

**Background/aim:**

*Serratia marcescens* which is a nosocomial pathogen, is naturally resistant to a wide spectrum of antibiotics, which makes the management of infections difficult. The aim of this study was to determine the *in vitro* susceptibilities of *S. marcescens* to ceftriaxone, ceftazidime, meropenem, amikacin, gentamicin, ciprofloxacin, and to compare the metabolic profiles of meropenem-resistant isolates under basal conditions and after exposure to sublethal concentrations of meropenem.

**Materials and methods:**

A total of 84 *S. marcescens* isolates were included from various samples. Genes for meropenem resistance were determined by polymerase chain reaction (PCR). Genetic similarities among isolates of *S. marcescens* were investigated by pulsed-field gel electrophoresis (PFGE). MIC changes of meropenem were investigated in the presence of the resistance-nodulation-cell division (RND) type pump inhibitor phenylalanyl-arginyl-β-naphthylamide (PAβN) and proton ionophore (uncoupler) carbonyl cyanide m-chlorophenylhydrazone (CCCP). A GC/MS-based metabolomics approach was implemented to determine the differentiation of metabolome structure. We examined the adaptive responses of isolates, characterized by resistance or susceptibility, under conditions of meropenem-induced stress.

**Results:**

The highest resistance rate was observed for ceftriaxone (27.6%). Amikacin was the most effective drug, with a resistance rate of 6.9%. Overall, 10 (11.9%) isolates were resistant to meropenem. Genotyping of β-lactamase genes revealed that *bla*_OXA-48_ was present in one isolate. In total, efflux pump activity was detected in four isolates. The GC/MS-based metabolomics analysis revealed alterations in nucleotide and pyrimidine metabolism, as well as in ATP-binding cassette (ABC) transporter pathways, between the meropenem-susceptible and meropenem-resistant groups.

**Conclusion:**

Understanding the metabolic profiles of *S. marcescens* could facilitate the development of novel diagnostic approaches and antimicrobial strategies in the ongoing global effort to combat meropenem-resistant *S. marcescens*.

## 1. Introduction

*Serratia marcescens* is a motile, uncommon gram-negative bacillus classified in the large family *Enterobacterales*. It has emerged in recent years as a cause of healthcare-associated infections such as meningitis, bacteremia, urinary infections, endocarditis, pneumonia, conjunctivitis, and wound infections, and has been isolated from outbreaks in neonatal intensive care units (NICUs) [1]. *S. marcescens* accounts for 2.2% of bloodstream infections, 2.8% of surgical site infections, and 3.6% of pneumonia cases in the USA. It has been reported to account for as much as 15% of hospital infections and is responsible for 5% of bloodstream infections in NICUs [2].

There are four principal mechanisms of resistance to β-lactam drugs in *S. marcescens*: i) production of inactivating enzymes (β-lactamases); ii) constitutive expression of efflux pumps; iii) low permeability of its outer membrane; and iv) alteration of penicillin-binding protein (PBP) targets [3]. The most common mechanism of resistance is β-lactamase production. *S. marcescens* may harbor chromosomal AmpC β-lactamase genes. Acquired resistance to extended-spectrum β-lactam antibiotics in *S. marcescens* is plasmid-encoded and confers resistance to third-generation cephalosporins. Moreover, *S. marcescens* has intrinsic resistance to ampicillin, amoxicillin, amoxicillin-clavulanate, ampicillin-sulbactam, and several narrow-spectrum cephalosporins. Chromosomal SME enzymes and plasmid-encoded IMP, VIM, NDM, and KPC enzymes have been reported in carbapenem-resistant *S. marcescens* [4].

Efflux pump families, which have been shown to exist in gram-negative bacteria to date, have also been identified in *S. marcescens* [5]. Among these, SdeXY, an RND-type efflux pump, has been associated with β-lactam resistance in this microorganism. However, the role of efflux pumps in acquired or adaptive carbapenem resistance in *S. marcescens* is not yet clear. Phenyl-arginine beta-naphthylamide (PAβN) and carbonyl cyanide 3-chlorophenylhydrazone (CCCP) are two compounds that inhibit the activity of RND type efflux pumps [6]. Significant decreases in antibiotic MICs detected in the presence of these efflux inhibitors are widely used to phenotypically demonstrate the role of efflux in antibiotic resistance [7].

State-of-the-art global metabolomics approaches are key for understanding the connections between antimicrobial resistance mechanisms (AMR) and microbial metabolism. Bacterial metabolic activity plays an essential role in cellular and cell-to-cell interactions. Therefore, these functions are associated with various AMR mechanisms. An emerging area of metabolomics allows for an in-depth investigation of bacterial metabolic processes using analytical mass spectrometry methods [8]. Metabolomic analyses of antimicrobial-resistant bacteria have been conducted in various species, including *Escherichia coli* [9], *Pseudomonas aeruginosa* [10], *Acinetobacter baumannii* [11], and *Klebsiella pneumoniae* [12]. However, there is limited literature available on the metabolomics of *S. marcescens* concerning the effects of drugs. This study focused on the genotypic and metabolic characterization of meropenem-susceptible and meropenem-resistant *S. marcescens* clinical isolates. Therefore, we characterized the resistance genes by PCR and compared the metabolic profiles of several meropenem-resistant clinical *S. marcescens* isolates under basal conditions and after exposure to sublethal concentrations of meropenem.

## 2. Material and methods

### 2.1. Bacterial isolates and identification

*S. marcescens* isolates (n = 84) were obtained from various clinical specimens in the Bacteriology Laboratory of Hacettepe University Hospital between 2011 and 2019. Matrix-assisted laser desorption/ionization time-of-flight mass spectrometry (MALDI-TOF MS; Bruker, Bremen, Germany) was used for bacterial identification.

### 2.2. Antimicrobial susceptibility testing

Minimum inhibitory concentrations (MICs) were determined by the broth dilution method according to the EUCAST (2020; v 10.0) guidelines. For broth microdilution tests (BMD), ceftriaxone, ceftazidime, meropenem, amikacin, gentamicin, and ciprofloxacin were supplied in powder form (Sigma-Aldrich, St. Louis, MO, USA). Reference isolates of *Escherichia coli* ATCC 25922 and *Pseudomonas aeruginosa* ATCC 27853 were included as quality controls.

### 2.3. Molecular detection of β-lactamase-encoding genes

The amplification of β-lactamase-encoding genes, including *bla*_KPC_, *bla*_NDM-1_, *bla*_IMP_, *bla*_VIM_, *bla*_SPM_, *bla*_AIM_, *bla*_OXA-48_, *bla*_GES_, and *bla*_SME-1_, which are associated with carbapenem resistance, was performed by PCR assays in all isolates. The relevant primers used for PCR testing are listed in Table 1 [13–15]. PCR conditions were applied according to the protocol provided [16]. Briefly, PCR was performed in a total volume of 25 μL. The reaction mixture was prepared using sterile distilled water, 1X PCR buffer, 0.2 mM dNTPs, 2.5 mM MgCl_2_, 0.25 U Taq polymerase, 20 pmol of each primer set, and 2.5 μL of template DNA. Amplification was carried out under the following thermal cycling conditions: 10 min at 94 °C, followed by 36 cycles consisting of 30 s at 94 °C, 40 s at 52 °C, and 50 s at 72 °C, with a final extension of 5 min at 72 °C. DNA fragments were analyzed by electrophoresis in a 2% agarose gel at 100 V for 1 h in 1X TAE buffer (40 mmol/L Tris–HCl [pH 8.3], 2 mmol/L acetate, 1 mmol/L EDTA) containing 0.05 mg/L ethidium bromide.

### 2.4. Pulsed-field gel electrophoresis (PFGE) typing

Genetic similarities among isolates of *S. marcescens* were investigated by PFGE as previously described [17]. Briefly, an overnight culture of bacteria was suspended in 2 mL of cell suspension buffer, mixed with an equal volume of 1.5% low-melting agarose, and distributed in a plug mold. Genomic DNA in agarose plugs was lysed in lysis buffer, washed, and digested with the SpeI restriction enzyme (New England Biolabs, Beverly, MA, USA; Thermo Scientific, Waltham, MA, USA). The Lambda PFG Ladder (New England Biolabs, Beverly, MA, USA) was used as a DNA size marker. Electrophoresis of digested DNA was performed using a pulsed-field electrophoresis system (CHEF Mapper/DR III; Bio-Rad Laboratories, Hercules, CA, USA).

### 2.5. Efflux pump activity assay

To determine whether an RND-type pump has an effect on carbapenem resistance, changes in meropenem MICs were investigated in the presence of RND-type efflux pump inhibitors (EPIs), PAβN, and CCCP [7]. At least a four-fold reduction in the presence of EPIs compared to basal MIC values was considered significant.

### 2.6. Metabolomics sample preparation

Following the determination of the meropenem susceptibility profiles of the isolates, for metabolomic experiments, meropenem-susceptible (CS) and meropenem-resistant (CR) *S. marcescens* isolates were selected from sterile specimens (blood, cerebrospinal fluid, pleural and peritoneal fluids, tissue biopsies). Sterile samples were exclusively selected to prevent contamination from bacteria present in nonsterile specimens and to ensure precise interpretation of metabolite data.

A GC/MS-based metabolomics approach to determine the differentiation of metabolome structure was implemented using the method described previously [18]. Briefly, meropenem-susceptible (CS-M) and meropenem-resistant (CR-M) isolates, either exposed or unexposed to a subinhibitory concentration of meropenem, were included. For meropenem-exposed groups, 0.5 × MIC of meropenem was used for each isolate, which was cultured on Luria–Bertani (LB) medium at 37 °C until the log phase was achieved. Following the incubation period, bacterial suspensions were centrifuged at 6000 rpm for 15 min. Cell pellets were then washed twice in sterile saline, and metabolites were extracted with a methanol–water (9:1, v/v) mixture. The mixture was then frozen and thawed three times in liquid nitrogen to disrupt the bacterial cell walls. The samples were then centrifuged at 15,000 rpm for 10 min, and the supernatants were evaporated in a vacuum centrifuge. Derivatization was performed by the sequential addition of 20 μL of methoxyamine solution in pyridine (20 mg/mL; Sigma Aldrich, St. Louis, MO, USA) at 30 °C for 90 min and 80 μL of N-methyl-N-trimethylsilyl trifluoroacetamide with 1% trimethylchlorosilane (MSTFA + 1% TMCS; Thermo Scientific, Waltham, MA, USA) at 37 °C for 30 min. Quality control (QC) samples were prepared using pooled samples. QC samples were prepared using the same experimental procedures as the test samples.

### 2.7. GC/MS analysis

The GC/MS-based metabolomics approach used to determine the differentiation of metabolome structure was implemented using the method described previously [18].

Metabolites were analyzed using GC–MS (Shimadzu GCMS-QP2010 Ultra; Shimadzu, Kyoto, Japan) with a DB-5MS stationary phase column (30 m + 10 m DuraGuard × 0·25 mm i.d. and 0·25 μm film thickness). Samples were injected in splitless mode, and the injection volume was adjusted to 2 μL. The oven temperature program was fixed at 70 °C for 1 min, then increased to 325 °C at a rate of 10 °C/min and held for 10 min at 325 °C. Total separation time was 37.5 min. Electron impact ionization was performed at 70 eV. Data acquisition was performed in full scan mode with a mass range of 50–650 m/z.

The raw MS data were evaluated using the MS-DIAL metabolomics platform [19]. Peak detection, deconvolution, and alignment processes were applied with default parameters. Fiehn retention index database was used for metabolite identification. The identification threshold was set at 60%.

The MetaboAnalyst 6.0 platform was used for statistical analysis. Principal component analysis (PCA) was used for general metabolome evaluation. The two-sample t-test was used to identify altered metabolites between experimental groups. In the statistical analysis, p < 0.05 and a fold change cutoff value (FC) > 1.25 were selected to identify altered metabolites between experimental groups.

The KEGG database was used for pathway analysis. The organism was selected as *S. marcescens*. In the analysis, at least two metabolites were selected for pathway identification.

## 3. Results

### 3.1. Bacterial isolates

A total of 84 *S. marcescens* isolates were included in the study. Susceptibility rates to antibiotics are shown in Table 2. The highest resistance rate was detected against ceftriaxone (27.6%). Amikacin had the lowest resistance rate (6.9%). Overall, 10 (11.9%) isolates were resistant to meropenem. Seventy percent (7/10) of meropenem-resistant *S. marcescens* isolates exhibited the multidrug-resistant (MDR) phenotype.

According to in vitro susceptibility to meropenem, isolates selected from sterile samples were categorized as meropenem-resistant (n = 6) and meropenem-susceptible (n = 6) for GC/MS metabolite analysis.

Genotyping of β-lactamase genes revealed that *bla*_OXA-48_ was present in only one isolate. In the BMD test performed to determine RND-type efflux pump activity on meropenem resistance, CCCP significantly reduced meropenem MICs in three isolates and PAβN in one isolate.

**Figure 1:**
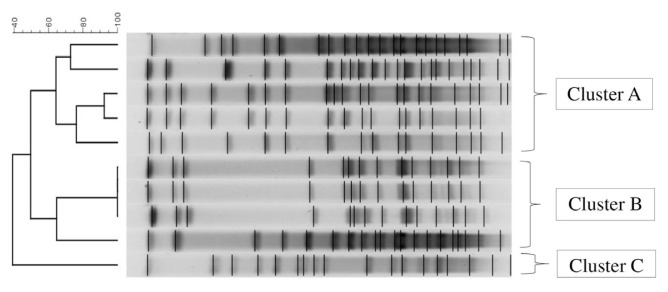
Dendrogram of PFGE profiles of meropenem-resistant *Serratia marcescens* isolates.

Eight pulsotypes were detected by PFGE. Isolates with genetic homologies were grouped into three clusters (A, B, and C) based on an approximate similarity threshold of 80% (Figure 1). MDR phenotype rates for Cluster A, B, and C were 4/5 (80%), 3/4 (75%) and 0/1 (0%), respectively. High resistance rates were observed for ceftazidime, ceftriaxone, and ciprofloxacin in isolates belonging to Cluster A. Similar to Cluster A, high resistance to ceftazidime, ceftriaxone, and ciprofloxacin was observed in isolates from Cluster B.

### 3.2. Metabolomic analysis

**Figure 2:**
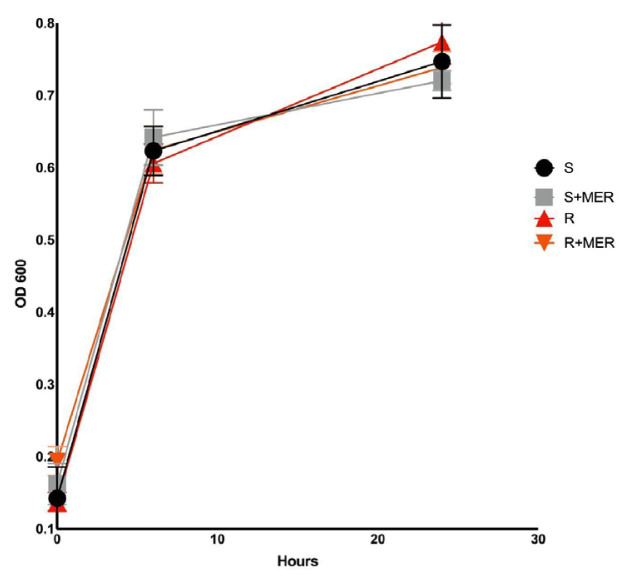
Growth curve of *Serratia marcescens* isolates with or without meropenem exposure.

**Figure 3:**
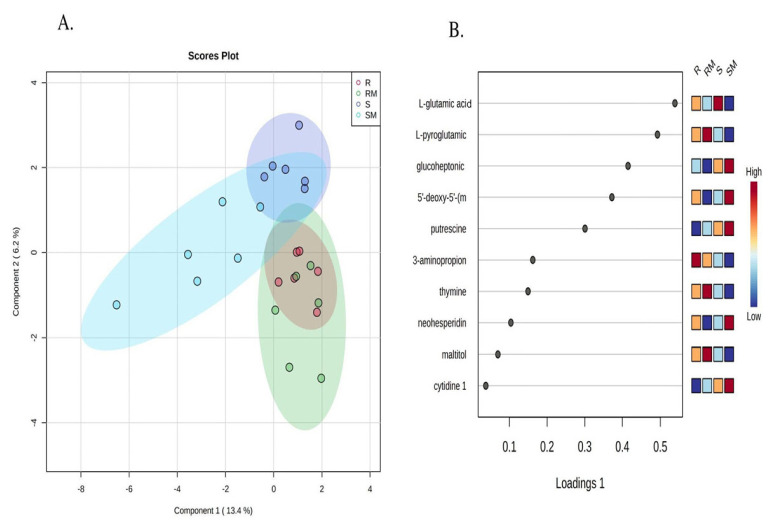
A) S-PLSDA analysis of experimental groups. B) VIP scores of metabolites. (S: susceptible isolates; R: resistant isolates; SM: meropenem-exposed susceptible isolates; RM: meropenem-exposured resistant isolates). Significant metabolites were identified by p < 0.05 and fold change (log_2_FC) > 1.25.

The bacterial growth curve was constructed by measuring absorbance at 600 nm at 0, 6, and 24 h to determine growth dynamics prior to sampling for metabolomics (Figure 2). In the metabolomics analysis (p < 0.05 and FC > 1.25), a total of 135 metabolites were identified by GC/MS. Results were evaluated in MetaboAnalyst platform to understand physiological changes of susceptible and resistant isolates under antibiotic stress condition. Sparse PLS-DA results showed that the metabolome structures of susceptible and resistant isolates were quite different from each other. Moreover, their responses to antibiotic-dependent stress factors were dramatically different, as shown in Figure 3. VIP scores of the susceptible and resistant experimental groups, their S-PLSDA analyses, and metabolites influenced by meropenem exposure are presented in Figures 3A and 3B.

**Figure 4:**
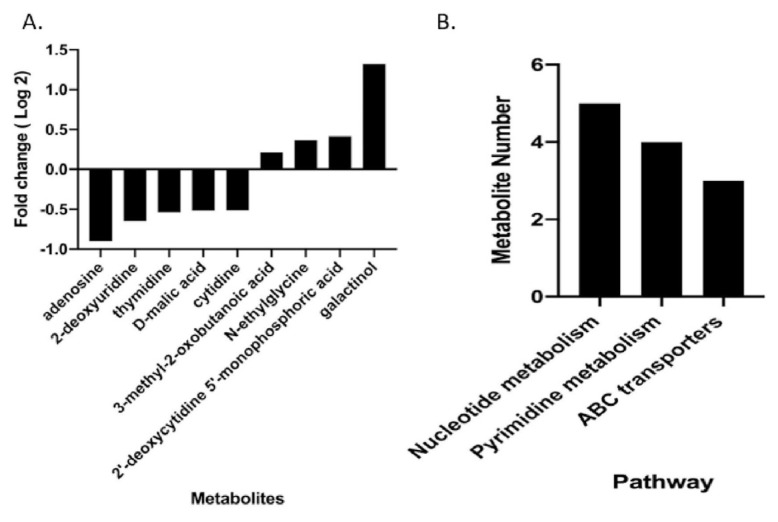
A) Altered metabolites with their fold changes (log_2_FC) between resistant and susceptible isolates. B) Altered biological pathways. Significant metabolites were identified by p < 0.05 and fold change (log_2_FC) > 1.25.

In the present work, we focused on molecular differences between resistant and susceptible isolates to understand the resistance mechanism at the metabolite level. We observed that nine metabolites were altered between the two experimental groups. The altered metabolites and their log_2_ fold changes between the two experimental groups are presented in Figure 4A. The altered biological pathways between resistant and susceptible isolates are shown in Figure 4B.

**Figure 5:**
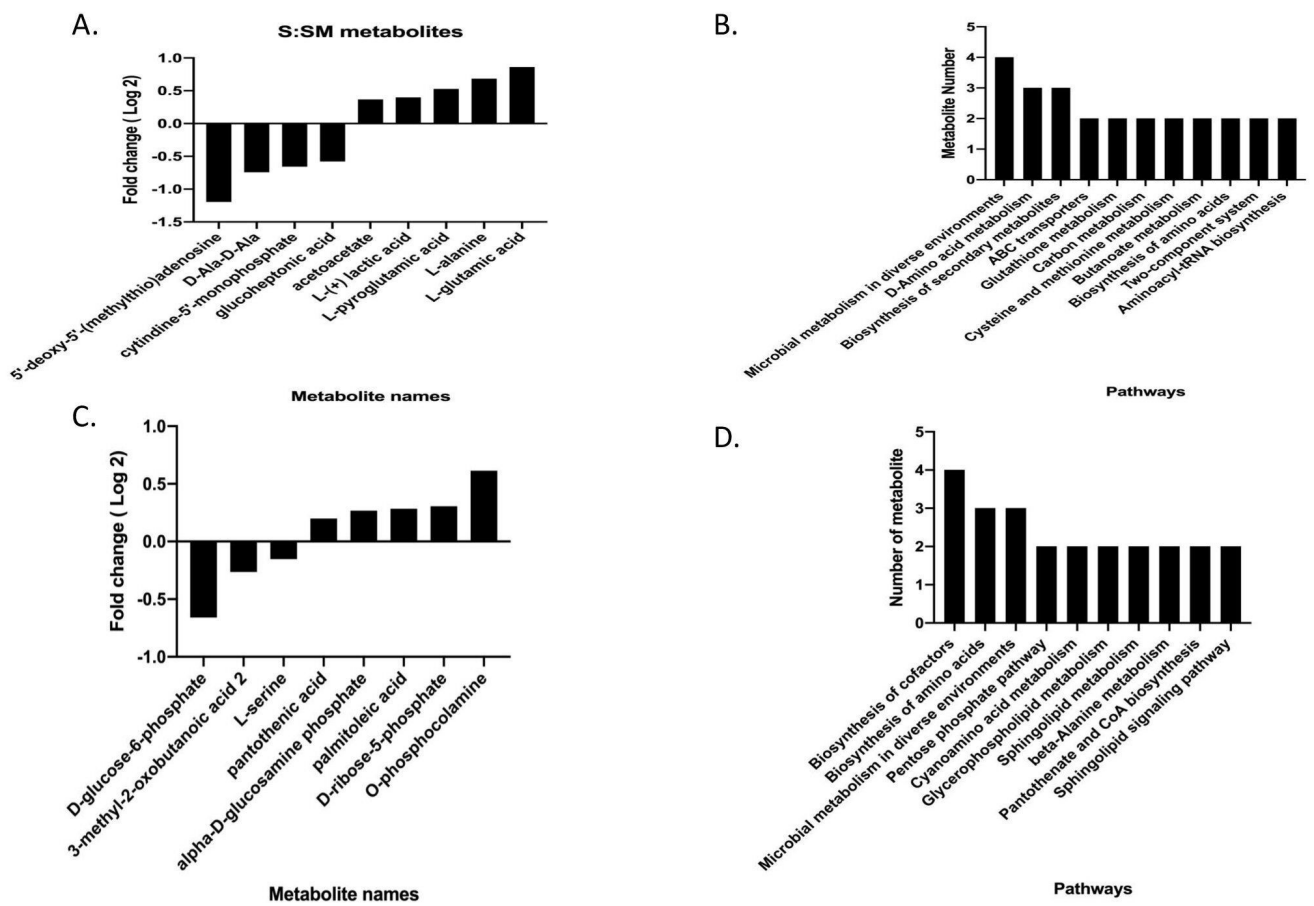
A) Altered metabolites in susceptible isolates under meropenem-induced stress. B) Altered biological pathways in susceptible isolates under meropenem treatment. C) Altered metabolites in resistant isolates under meropenem-induced stress. D) Altered biological pathways in resistant isolates under meropenem treatment. Significant metabolites were identified by p < 0.05 and fold change (log_2_FC) > 1.25.

We also analyzed the metabolome structure of resistant and susceptible isolates under meropenem-induced stress conditions. Altered metabolites and related pathways for resistant and susceptible isolates are shown in Figures 5A–5D.

## 4. Discussion

*S. marcescens* is a serious cause of nosocomial infections. Outbreaks caused by *S. marcescens* have been reported in several intensive care units worldwide [20]. Revealing the relationship between *S. marcescens* isolates and potential antibiotic resistance genes through comprehensive approaches is significant for elucidating the epidemiology of outbreaks caused by *S. marcescens*. In our study, we aimed to highlight the potential resistance mechanisms involved in meropenem-resistant isolates using phenotypic, genotypic, and metabolomic approaches.

This study demonstrated that ceftriaxone and ciprofloxacin resistance rates were high among *S. marcescens* isolates. The prevalence of ceftriaxone resistance among *S. marcescens* isolates varies widely, from low in the USA (10.8%) and the Mediterranean region (11.3%) [21] to high in Taiwan (26.8%) [22], Poland (39%) [23], and China (22.5%) [24]. Globally, ceftazidime resistance rates (3.7%–9.0%) remain low compared to those of ceftriaxone among *S. marcescens* isolates [24]. We found the resistance rate of ceftazidime (17.4%) to be lower than that of ceftriaxone (27.6%). In various reports from different countries, resistance to ciprofloxacin in *S. marcescens* has been reported to range from 3.2% to 36% [3, 23, 25]. In this study, the ciprofloxacin resistance rate (26.5%) was similar to that reported in recent studies.

*S. marcescens* isolates are frequently susceptible to aminoglycosides; nevertheless, recent reports demonstrate increasing resistance to gentamicin and amikacin [22]. Bertrand and Dowzicky reported amikacin susceptibility between 75% and 100% in *S. marcescens* isolates from intensive care units in different geographic regions [26]. In a multicenter study by Sader et al., amikacin and gentamicin resistance rates of *S. marcescens* isolates were reported as 0.4% and 0.8% in the USA, and 2.3% and 2.5% in the Mediterranean region, respectively [21]. In a study on *S. marcescens* isolates reported from Türkiye, amikacin resistance was reported as 10% and gentamicin resistance as 25% [27]. In our study, amikacin had the lowest resistance rate.

At present, carbapenems are among the most effective antibiotic groups against *Enterobacterales*. According to a report published by the SENTRY Antimicrobial Surveillance Program, the meropenem resistance rate of *S. marcescens* isolates between 2009 and 2012 was 0.4% in the USA, while all *S. marcescens* isolates were reported as susceptible to meropenem in the Mediterranean region [21]. In this study, we detected meropenem resistance in 10 (11.9%) *S. marcescens* isolates. One of the common mechanisms of *S. marcesc*ens resistance to carbapenems is the production of plasmid-encoded carbapenemase types such as *bla*_OXA_, *bla*_IMP_, *bla*_VIM_, and *bla*_KPC_ [28–30]. In a study by Ymaña et al. investigating *bla*_IMP_, *bla*_KPC_, *bla*_NDM_, *bla*_OXA-48_, and *bla*_VIM_ carbapenemase genes in *S. marcescens* isolates obtained from intensive care unit specimens, the coexistence of *bla*_KPC_ and *bla*_NDM_ genes was reported in two extensively drug-resistant (XDR) isolates [31]. In our present study, that *bla*_OXA-48_ was detected in only one isolate. This finding suggests the presence of noncarbapenemase mechanisms of resistance in meropenem-resistant isolates.

The role of efflux pumps in acquired or adaptive carbapenem resistance in *S. marcescens* is not yet clear [20]. In this study, we investigated the effects of PAβN and CCCP on the meropenem MICs of the isolates. CCCP in three isolates and PAβN in one isolate significantly reduced meropenem MICs. These results strongly suggest that an RND-type efflux pump plays an active role in the meropenem resistance of the isolates. In this respect, our study is the first to demonstrate the active role of an RND-type efflux pump in carbapenem resistance in *S. marcescens*. The specific roles of RND-type efflux pumps, especially SdeXY, in carbapenem resistance should be investigated in detail.

### 4.1. Metabolomics

In the present study, we focused on clarifying the resistance mechanisms exhibited by *S. marcescens* against meropenem. We conducted a comprehensive analysis of the metabolome structures within both meropenem-resistant and susceptible isolates. Furthermore, we examined the adaptive responses of isolates—characterized by resistance or susceptibility—under meropenem-induced stress conditions. This study, which investigated the effect of meropenem on the metabolome of *S. marcescens* isolates, revealed differences in metabolite profiles between groups, including CS and CR *S. marcescens* isolates exposed to sub-MIC meropenem (CR-M and CS-M).

#### 4.1.1. Differences in the metabolome structures of carbapenem-resistant and carbapenem-susceptible isolates

Metabolomics is an emerging tool for understanding physiology of organisms under different conditions. Especially in microbiology, metabolomics can offer essential information for understanding bacterial behavior under different stress conditions. A differentiated metabolome structure indicates new resistance targets and pathways. In the present study, we used metabolomics to elucidate the resistance mechanism of *S. marcescens* against carbapenems by comparing the metabolome structure of resistant and susceptible isolates.

PLS-DA analysis showed that the general metabolome structure shifted dramatically between CS and CR isolates. This result indicates that the metabolite profile could be one of the key factors in the resistance process.

After applying p < 0.05 and FC > 1.25, we observed that nine metabolites were altered between the two groups, and these metabolites were involved in various pathways.

Remarkably, nucleotide and pyrimidine metabolism, as well as ABC transporters, were the pathways that showed significant differences between the CS and CR groups. Considering the involvement of these three metabolic pathways, it can be predicted that the concentrations of nucleic acid metabolites such as adenosine, thymidine, deoxycytidine monophosphate, cytidine, and deoxyuridine differ between the groups. Previous metabolomic studies indicate that purines play a crucial role in a variety of processes, including energy metabolism, cell signaling, and gene coding. Pyrimidine metabolism is associated with repair and survival functions under environmental stress in bacteria [32–35]. Consequently, disrupting the metabolic equilibrium of purines and pyrimidines adversely affects the physiological activities of bacteria, such as DNA synthesis and metabolism.

The compound 3-methyl-2-oxobutanoic acid is a precursor mainly involved in the biosynthetic pathways of branched-chain amino acids (BCAAs) such as valine, leucine, and isoleucine, as well as in pantothenate (vitamin B5) and CoA biosynthesis. The significance of BCAAs in bacterial physiology arises from their integration into central metabolism, their necessity for protein synthesis, and their essential role in environmental adaptation [36]. Beyond stimulating protein synthesis and facilitating growth in gram-positive bacteria deficient in BCAAs, investigations involving *Listeria monocytogenes* have revealed its inability to furnish the requisite content of branched-chain fatty acids (BCFAs) for shielding against host defenses directed at the bacterial membrane [37,38]. In this study, we observed that 3-methyl-2-oxobutanoic acid was upregulated in the resistant group.

Another critical metabolite, sarcosine (N-methylglycine), which is related to metabolites associated with the pyruvate pathways, is prevalent in various environments inhabited by pseudomonads and is predominantly encountered as an intermediary compound in the metabolic pathways of choline, carnitine, creatine, and glyphosate [39]. In the pulmonary environment, *Pseudomonas aeruginosa* obtains choline through the action of the virulence factors phospholipase C (PlcH) and phosphorylcholine phosphatase on phosphatidylcholine [40, 41]. In the present work, upregulated levels of sarcosine were observed in the resistant group, which correlates with prior studies.

#### 4.1.2. Bacterial behavior and adaptation process of *S. marcescens* under meropenem treatment

In the present work, we also evaluated the adaptation of *S. marcescens* isolates (carbapenem-resistant and susceptible) under meropenem treatment using metabolomics analysis. PLS-DA analysis showed that the metabolome structures of meropenem-treated groups were quite different from those of the control groups in both CR and CS isolates. This indicates an adaptation process under stressful conditions. We used altered metabolites and related pathways to explain this adaptation process.

#### 4.1.3. Comparison of CR and CR-M metabolic profiles

In the study, CR groups were compared with CR-M groups, which were exposed to meropenem at sub-MIC concentrations. Several metabolites, including L-serine, D-ribose-5-phosphate, 3-methyl-2-oxobutanoic acid, ethanolamine phosphate, pantothenate, and β-D-glucose-6-phosphate, were differentiated between the two groups. These metabolites are involved in pathways such as secondary metabolite biosynthesis, cofactor and amino acid biosynthesis, adaptation to diverse environments, cyanoamino acid metabolism, pentose phosphate pathway, sphingolipid metabolism, and glycerophospholipid metabolism. Serine is phosphorylated by kinases and participates in the biosynthesis of purines and pyrimidines in bacteria. Moreover, it is a precursor of several amino acids, such as glycine, cysteine, and tryptophan, which is involved in cell signaling mechanisms [42, 43]. In the study of Yang et al. associated with the bacterial zoonosis agent *Edwardsiella tarda*, it was predicted that exogenous serine is dependent on glutathione metabolism, which down-regulates reactive oxygen species to reduce immune responses [44]. According to the metabolic pathway descriptions in KEGG, D-ribose-5-phosphate and β-D-glucose-6-phosphate are metabolized through glycolysis, and the pentose phosphate pathways^1^

Previous metabolomics studies with *Acinetobacter baumannii* and *Pseudomonas aeruginosa* revealed that the levels of pentose and phosphate pathways metabolites were significantly altered by various antibiotic combination treatments [45–47]. Glycerophospholipids are critical components of the dual-membrane envelope of gram-negative bacteria [48]. Alterations in glycerophospholipid levels and membrane composition occur under environmental stresses, such as the presence of antibiotics in bacterial metabolism [49].

#### 4.1.4. Comparison of CS and CS-M metabolic profiles

Two metabolic pathways—alanine metabolism and glutamate metabolism—were significantly altered after exposure to meropenem in the CS group. These pathways are involved in bacterial metabolic processes such as the ABC transport system; D-amino acid, glutathione, carbon, cysteine, methionine, and butanoate metabolism; and amino acid and aminoacyl-tRNA biosynthesis. Mahamad et al. integrated metabolomic and transcriptomic analysis of the synergistic effect of polymyxin-rifampicin combination against *Pseudomonas aeruginosa* showed that the combination significantly altered alanine biosynthesis [50].

This study has several limitations. First, the metabolomic analysis was limited to sterile-site isolates, excluding other specimen types. Second, although distinct metabolic differences were identified between meropenem-resistant and susceptible *S. marcescens* isolates, the absence of transcriptomic, proteomic, or whole-genome sequencing data limits mechanistic interpretation.

## 5. Conclusion

The study revealed significant metabolic and genomic differences between CR and CS *S. marcescens* strains. As carbapenem resistance in the isolates described here was not associated with the production of a carbapenemase, the mechanism of resistance is the subject of further investigation. We investigated significant changes in cell envelope biosynthesis, glycerophospholipid metabolism, the pentose phosphate pathway, energy metabolism, and nucleotide and amino acid metabolism in meropenem-resistant *S. marcescens* isolates. Our findings provide valuable insights into meropenem resistance in *S. marcescens*, which may aid in optimizing the clinical repositioning of meropenem.

## Figures and Tables

**Table 1:** Primers used for PCR amplification of β-lactamase-encoding genes.

Primer	Sequence (5′–3′)	Gene	Product size (bp)
**KPC**	F-CGTCTAGTTCTGCTGTCTTG R-CTTGTCATCCTTGTTAGGCG	*bla*_KPC_	798 232
**NDM-1**	F-GGTTTGGCGATCTGGTTTTC R-CGGAATGGCTCATCACGATC	*bla*_NDM-1_	699
**IMP**	F-GGAATAGAGTGGCTTAAYTCTC R-GGTTTAAYAAAACAACCACC	*bla*_IMP_	232
**VIM**	F-GATGGTGTTTGGTCGCATA R-CGAATGCGCAGCACCAG	*bla*_VIM_	390
**SPM**	F-AAAATCTGGGTACGCAAACG R-ACATTATCCGCTGGAACAGG	*bla* *_SPM_*	271
**AIM**	F-CTGAAGGTGTACGGAAACAC R-GTTCGGCCACCTCGAATTG	*bla* *_AIM_*	322
**OXA**	F-GCGTGGTTAAGGATGAACAC R-CATCAAGTTCAACCCAACCG	*bla* *_OXA-48_*	438
**GES**	F-ATGCGCTTCATTCACGCAC R-CTATTTGTCCGTGCTCAGG	*bla* *_GES-1_*	860
**SME**	F-GTGTTTGTTTAGCTTTGTCGGC R-GCAATACGTGATGCTTCCGC	*bla* *_SME_*	801

**Table 2:** *In vitro* susceptibility of *Serratia marcescens* isolates to antibiotics (n = 84).

	Antibiotics	MİK range*	MİK_50_*	MİK_90_*	S (%)	R (%)
**β-lactams**	Ceftriaxone	≤0.125 to >256	0.25	128	72.4	27.6
	Ceftazidime	≤ 0.125 to >256	0.125	32	82.6	17.4
	Meropenem	≤ 0.125 to 256	0.125	16	88.1	11.9
**Aminoglycosides**	Amikacin	≤ 0.125 to 256	1	8	93.1	6.9
	Gentamicin	≤ 0.015 to 32	0.25	8	86.2	13.8
**Fluoroquinolones**	Ciprofloxacin	≤ 0.015 to 32	0.06	4	73.5	26.5

* mg/L, S: susceptible, R: resistant.

## Footnotes

1 Kyoto Encyclopedia of Genes and Genomes (KEGG) [online]. Website: https://www.genome.jp/kegg/ [accessed 09 August 2025]
